# Polymorphism in *IGFALS* gene and its association with scrotal circumference in Hu lambs

**DOI:** 10.1080/10495398.2023.2295928

**Published:** 2024-01-04

**Authors:** Zongwu Ma, Weimin Wang, Deyin Zhang, Xinji Wang, Shirong Li, Liming Zhao, Yukun Zhang, Yuan Zhao, Xiaolong Li, Changchun Lin, Jianghui Wang, Jiangbo Cheng, Dan Xu, Xiaobin Yang, Yongliang Huang, Panpan Cui, Jia Liu, Xiwen Zeng, Rui Zhai, Zhiqiang Huang, Xiuxiu Weng, Xiaoxue Zhang

**Affiliations:** a College of Animal Science and Technology, Gansu Agricultural University, Lanzhou, Gansu, China; b The State Key Laboratory of Grassland Agro-Ecosystems, College of Pastoral Agriculture Science and Technology, Lanzhou University, Lanzhou, Gansu, China; c Minqin County Animal Husbandry and Veterinary Workstation, Minqin, Gansu, China

**Keywords:** Scrotal circumference, SNP, *IGFALS*, Hu sheep

## Abstract

Scrotal circumference is an important reproductive index of breeding rams, which has a high genetic correlation with ejaculation volume and semen quality. In this study, the scrotal circumference of 1353 male Hu sheep at different stages of development was measured and descriptive statistical analysis was performed. The results showed that the coefficient of variation of scrotal circumference at each stage was greater than 10%, and its heritability were moderately to high, ranging from 0.318 to 0.719. We used PCR amplification and Sanger sequencing to scan the polymorphisms of the *IGFALS* gene, and performed association analysis with the circumference of the scrotum at different stages. We identified a synonymous mutation g.918 G > C in exon 1 of the *IGFALS* gene, and this mutation was significantly associated with scrotal circumference at 100, 120, 140, 160 and 180 days (*p* < 0.05). Therefore, *IGFALS* gene polymorphism can be used as a molecular marker affecting scrotal circumference of Hu sheep, which can provide a reference for future molecular marker-assisted selection of scrotal circumference in sheep.

## Introduction

Sheep (*Ovis aries*) are one of the earliest domesticated animals in China and play a very important role both economically and culturally. With the continuous improvement of people’s living standards, mutton has become popular because of its unique texture and flavor. As one of the important livestock in China, Hu sheep have the characteristics of high reproductive performance, fast growth and strong adaptability, especially famous for early sexual maturity, year-round estrus, large lambing numbers, and strong lactation ability.^1^^,^^2^ Although the number of sheep in our country is very large, because of the large population base, the number of sheep per capita is very small. Therefore, it is particularly important to speed up molecular breeding and breed new varieties of sheep with high fertility.

Scrotal circumference is an important indicator and reliable method for evaluating the reproductive capacity of rams. The size of the scrotal circumference can directly represent the size of the testicles. In previous studies, it was found that the circumference of the scrotum is closely related to testicular size and sperm production.^3–5^ Some researchers have found that Scrotal circumference or testicular weight were positively correlated with number of sperm obtained by frequent ejaculation.^4^^,^^6–9^ Scrotal circumference is a highly genetic trait and its age-related changes can be used as a measure of puberty. The relationship between sexual maturity and scrotal circumference has also been reported in male goats.^10^^,^^11^ Menegassi et al.^12^ found that bulls scrotal circumference was associated with an increase in testicular weight and live weight. Insulin like peptide 3 (INSL3) is considered a specific biomarker of puberty testes,^13–16^ and in studies of male goats, it was found that the correlation between scrotal circumference and INSL3 concentration is higher than that of testosterone. The average scrotal circumference of male goats also shows significant changes before and after puberty.^17^ The measurement of scrotal circumference plays an important role in the onset of puberty, total semen volume, semen quality, pathological status of the reproductive system, and reproductive status of breeding bulls.^18^ A study found that bulls with a scrotal circumference of 27.9 cm^19^ or 50 million sperm produced at the first ejaculation with at least 10% of forward motility^20^ can be considered to have reached puberty. Compared with adolescent bulls, the scrotal circumference and semen parameters of bulls significantly increase after puberty, and the sperm density of bulls after puberty is positively correlated with the scrotal circumference.^21^ In sheep studies, it has been confirmed that breed and season have a significant impact on scrotal circumference and semen characteristics.^22–24^ Studies have found that Katahdin and Dubo rams have a higher scrotal circumference than Blackbell and Pelibey rams.^25^ However, there are few reports on the periodic changes in the circumference of the scrotum in sheep. Scrotum is the external protective layer of the testis, which can regulate the temperature of the cystic cavity and promote the generation and development of sperm. The increase in testicular temperature will reduce sperm motility, concentration, normal morphology, acrosome integrity and chromatin stability.^26^

The screening of the main genes affecting reproductive ability is very important for sheep breeding. Insulin-like growth factor-binding protein acid-labile subunit (*IGFALS*) gene was first successfully cloned in a mice.^27^ The *IGFALS* gene encodes an acid-labile subunit (ALS) protein that binds to IGF-I and IGFBP-3 to form a ternary complex in the circulation that regulates growth and development and other physiological processes.^28^ Human ALS deficiency is characterized by slow growth and development, the secretion of GH is the same as or even higher than normal, the levels of IGF-1 and IGFBP-3 are significantly reduced, and they are still low after treatment.^29^ This is caused by homozygous or compound heterozygous inactivation mutation of *IGFALS* gene.^30^ *IGFALS* gene have been identified in humans,^31^ mice,^27^ sheep,^32^ rats^33^ and pigs,^34^ and plays an important role in the growth and development of mammals. Several studies have shown that polymorphisms in *IGFALS* are associated with growth traits in Makouei, Ghezel sheep, and cattle.^28^^,^^35^ In the study of S. Li et al.,^34^ *IGFALS* was found to be expressed in multiple tissues of the pig testis, ovaries, epididymis, liver, lung, and prostate. However, there are no reports on the association of this gene with the scrotal circumference in sheep. Therefore, the aim of this study was to investigate the relationship between the *IGFALS* gene SNP and scrotal circumference in Hu sheep.

## Materials and methods

### Statement of ethics

All experimental procedures in this study were approved by the Animal Care and Use Committee of Biological Research of Gansu Province, China. And the experimental protocol and sample collection were approved by the Ethics Committee of Gansu Agricultural University (permit number for conducting animal experiments: NO. 2012–2-159).

### Animal management

A total of 1662 male Hu sheep used in this study were selected from Jinchang Zhongtian Sheep Industry Co. Ltd., Gansu Zhongsheng Huamei Sheep Industry Development Co. Ltd., Gansu Sanyangjinyuan Husbandry Co. Ltd., Shandong Runlin Sheep Industry Co. Ltd, and Wuwei Pukang Sheep Industry Co. Ltd. All lambs were weaned at 56 days of age, and routine immunization before weaning was performed by professional workers. After weaning, all lambs were transferred to Minqin Defu Agriculture Co. Ltd (Gansu, China) for centralized feeding and management. The feeding of experimental animals included a 2-week adaptation period, a 10-day pre-testing period, and a 100-day experimental period. In the adaptation period, the proportion of feed in the diet was gradually reduced, and the proportion of pellet feed was gradually increased by 7.1% until reaching 100%. During the feeding period, all lambs were raised in separate enclosures (0.8 × 1 m) and fed pellet feed (Gansu Runmu Bioengineering Co., Ltb). The experimental animals were in a good ecological environment and perfect management, and were guaranteed to freely eat and drink water.

### Data recording, sample collection and DNA extraction

Scrotal circumference was measured and recorded on the morning of each growth stage (the 100th, 120th, 140th, 160th and 180th days) using a clearly graduated tape measure. The greatest SC of each lamb was measured using a clearly graduated tape, after pushing the testes firmly into the scrotum.^10^ On the last day of the experiment, blood samples (5 mL) were collected from each sheep by jugular vein sampling. DNA was extracted according to the instructions of the EasyPure Blood Genomic DNA Kit (TransGen Biotech, Beijing, China). DNA concentration and purity are measured by ultra-micro spectrophotometer. Qualified DNA was stored at −20° C for subsequent experiments. Finally, the blood DNA of 1353 Hu sheep was successfully extracted.

### Polymerase chain reaction and genotyping

Firstly, we used Oligo 7.0 and Primer 5 software to design a pair of specific primers containing partial exon 1 of the *IGFALS* gene to amplify an 848 bp fragment by referring to the reference sequence of *IGFALS* gene (GenBank Accession No. NC_040275.1) in NCBI database. Primer information is shown in Table 1. Ten Hu sheep mixed DNA samples were randomly selected from the experimental population for PCR amplification, and then the amplified products were sequenced to detect single nucleotide polymorphisms (SNPs) in *IGFALS* gene of sheep. PCR amplification was performed in a 35 µl reaction mixture. The reaction mixture is made up of 17.5 µl of 2× TSINGKE Master Mix (TSINGKE Biological Technology, Beijing, China), 14 µl of ddH_2_O, 1.4 µl of template DNA and 1.12 µl of primers (forward and reverse primers). The PCR thermal cycle is programmed as follows: pre-denaturation at 94 °C for 5 min, deformation at 94 °C for 30 s, annealing at 61.4 °C for 30 s, extension at 72 °C for 30 s and 35 cycles and a final extension at 72 °C for 5 min. SNPs in *IGFALS* gene were detected by sequencing the target fragment, and genotyping of all experimental individuals was performed using competitive allele-specific FRET-based PCR (KASPar) assays (LGC Genomics, Hoddesdon, UK). Primer information for KASPar genotyping is shown in Table 2.

**Table 1.** Primer pairs designed for sheep *IGFALS.*

Primer name	Primer sequences (5′–3′)	GenBank accession number	Annealing temperature (°C)	Amplicon length (bp)
*IGFALS*-F *IGFALS*-R	TGACGGCACCCAGCTAACTCA TCCTCCCCTGCTCATCCCA	NC_040275.1	61.4 °C	848 bp

**Table 2.** Primer pairs designed for KASPar assay.

Gene	Primer	Primer sequence (5′–3′)
*IGFALS*	Primer_AlleleX	GAAGGTGACCAAGTTCATGCTTGATGGCAGGCAACGCTTCAC
	Primer_AlleleY	GAAGGTCGGAGTCAACGGATTTGATGGCAGGCAACGCTTCAG
	Primer_Common	TTTACAGGAGATGACAACCCACTGG

### Statistical analysis

Finally, 1353 individuals had genotypic and phenotypic data. Based on genotyping results, the genetic index is calculated directly for *IGFALS* gene.^36^ The statistical software SPSS (version 25.0) was used to analyze the associations between genotypes and scrotal circumference in sheep. An adjusted multivariate linear model was established as follows:

$$Y_{\text{ijlnk}}=µ+G_{i}+F_{j}+F_{l}+M_{n}+S_{k}+\varepsilon_{\text{ijlnk}}$$

where *Y_ijlnk_* is the observation of the scrotal circumference traits, *µ* is a vector of mean, *G_i_* is the fixed effect of ith genotype, *F_j_* is the fixed effect due to jth farm (*j* = 1, 2, 3, 4, 5, 6), *F_l_* and *M_n_* represent the family effect, *S_k_* represents the effect of season (*k* = winter, summer), and *ɛ_ijlnk_* was random residuals. Significance of phenotypic means was tested by Duncan’s test and Tukey’s test. There was statistical significance at *p* < 0.05, and the results were expressed as mean ± standard error.

In this study, genetic parameters for scrotal circumference in bearded sheep were estimated using Hiblup software (https://www.hiblup.com/tutorials). To provide reference for Hu sheep breeding, a model that explains the additive effects and residuals in the model was fitted, as follows.

$$Y=X\beta +Z_{a}a+e$$

$$V\left(a\right)=A\sigma^{2}{}_{a}\;V(e)=I\sigma^{2}{}_{e}$$

*Y* is the record vector, *β* and e are the vectors of fixed effects and residual effects, respectively, *X* is the correlation matrix, and *I* denotes the unit matrix, *σ^2^_a_* and *σ^2^_e_* are the additive variance and residual variance, respectively.

## Results

### Scrotal circumference

**Figure 1.**
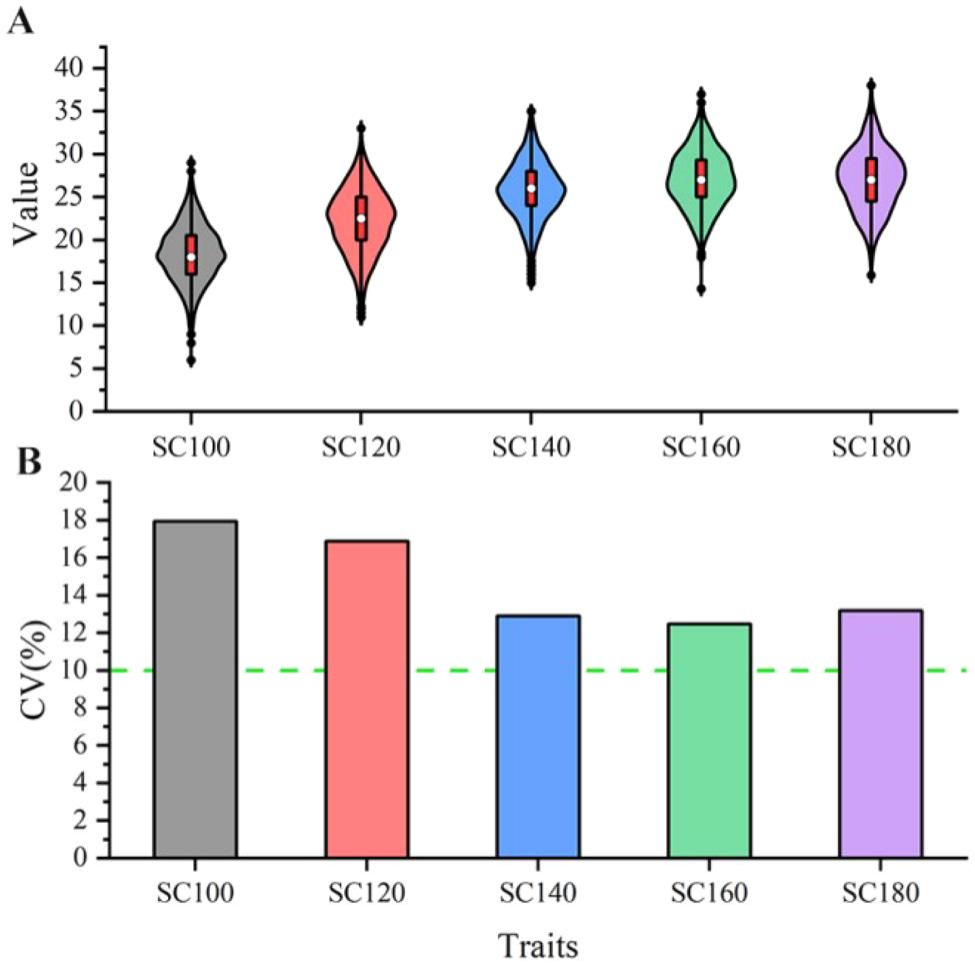
Descriptive statistics of scrotal circumference size at different stages. (A) The violin plot of scrotal circumference. (B) The coefficient of variation of scrotal circumference.

The scrotal circumference of 1353 male Hu sheep were measured on days 100, 120, 140, 160 and 180, respectively. The statistical results showed that the scrotal circumference at these five stages was 18.41 (± 3.30), 22.21 (± 3.75), 25.76 (± 3.32), 27.05 (± 3.37), and 27.02 (± 3.56), respectively, and the coefficient of variation of scrotal circumference in different stages was more than 10% (Fig. 1). The variance components and heritability estimate for scrotal circumference at different stages are shown in Table 3. The heritability of scrotal circumference at 100, 120, 140,160 and 180 days was 0.719 (± 0.116), 0.578 (± 0.126), 0.318 (± 0.123), 0.444 (± 0.126) and 0.4781 (± 0.186) for Hu sheep, respectively, which were of medium to high heritability.

**Table 3.** Estimation of scrotal circumference variance components and heritability at different stages.

Items	*σ*^2^*_a_* (±se)	*σ*^2^*_e_* (±se)	h_2_ (±se)
Scrotal circumference100	6.120 ± 1.117	2.397 ± 0.957	0.719 ± 0.116
Scrotal circumference120	6.016 ± 1.420	4.395 ± 1.251	0.578 ± 0.126
Scrotal circumference140	2.856 ± 1.137	6.112 ± 1.058	0.318 ± 0.123
Scrotal circumference160	4.245 ± 1.274	5.320 ± 1.153	0.444 ± 0.126
Scrotal circumference180	4.691 ± 1.934	5.119 ± 1.742	0.4781 ± 0.186

*Note*: *σ*^2^*_a_*: additive effect variance component; *σ*^2^*_e_*: residual variance component; h_2_: heritability.

### SNP of IGFALS gene

**Figure 2.**
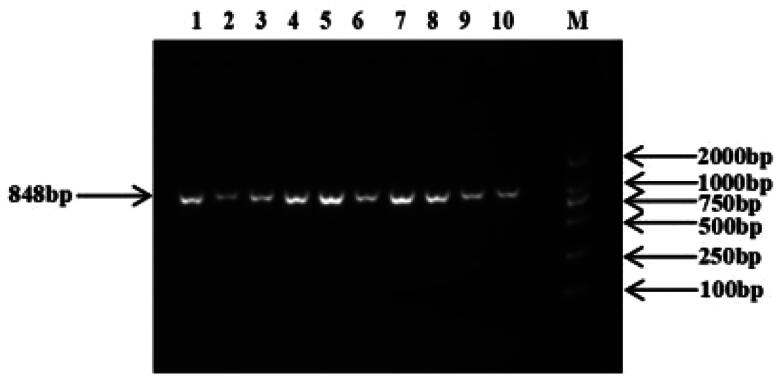
PCR amplification of the target fragments of the ovine *IGFALS* genes. *Note*: M: DL2000 DNA Marker; 1–10: PCR products

**Figure 3.**
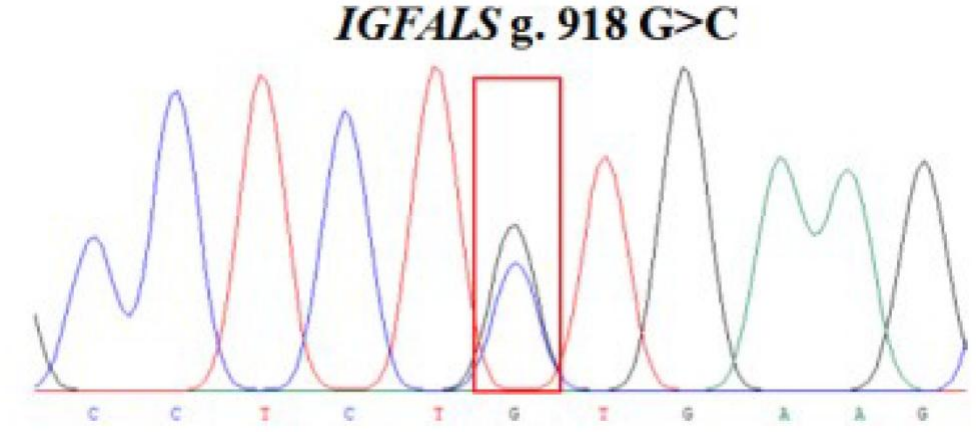
Image of the sequencing peaks of sheep *IGFALS* g.918 G > C loci.

**Figure 4.**
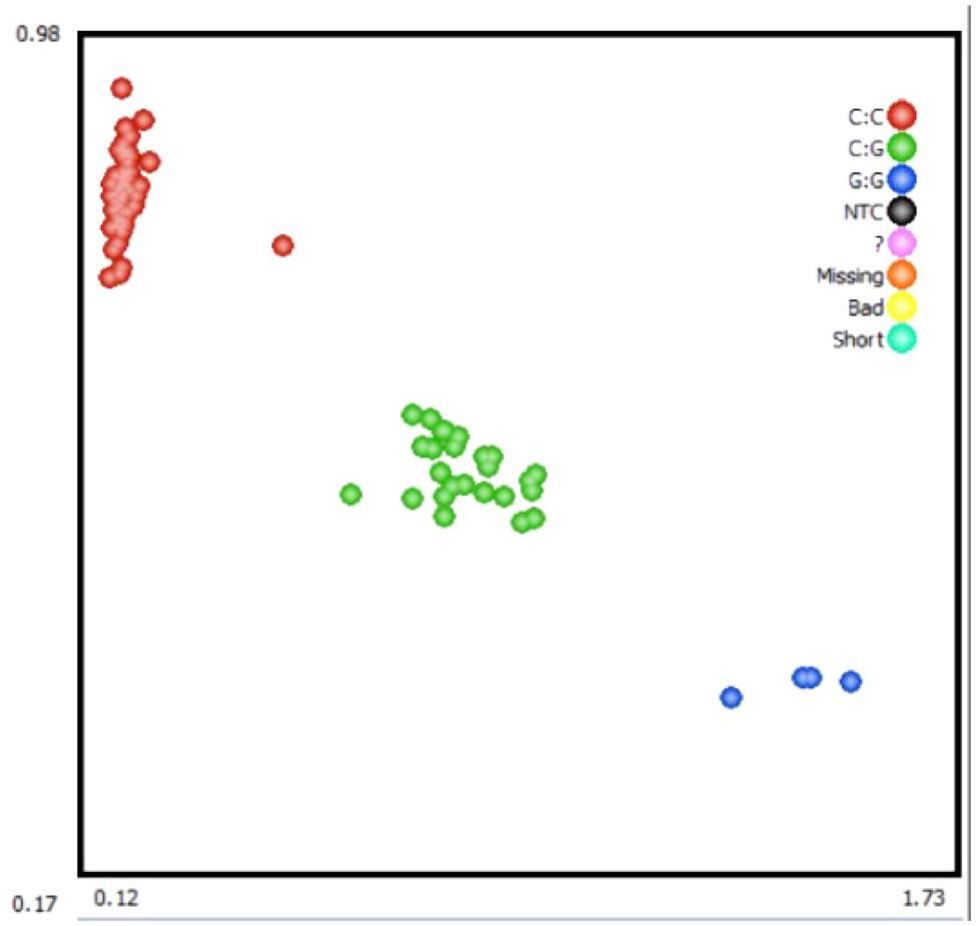
Kaspar–based single nucleotide polymorphism (SNP) genotyping of sheep *IGFALS* g.918 G > C.

The 848 bp fragment of *IGFALS* gene was successfully amplified by specific primers from 10 extracted DNA samples (Fig. 2). By sequencing the PCR product, a SNP (G/C) was identified at position 918 in exon 1 of the *IGFALS* gene **(**Fig. 3**)**. To determine the polymorphism of the *IGFALS* gene, KASPar assays were used to genotype the SNP at g.918 G > C of *IGFALS*. Three genotypes (CC, CG and GG) were detected after genotyping **(**Fig. 4**)**.

### Genetic indexes of IGFALS gene

The results of the genetic index analysis of the *IGFALS* gene are shown in Table 4. The results showed that the *IGFALS* gene was in a low level of heterozygosity in sheep population (0 < PIC < 0.25). In the experimental population, the CC genotype had the highest frequency of 0.73 among the three genotypes, so it was the main dominant genotype. C allele frequency is greater than G allele frequency and is the dominant allele. The *IGFALS* gene was in Hardy-Weinberg equilibrium in the experimental population.

**Table 4.** Genotype frequency, allele frequency, and genetic diversity of *IGFALS* g.918G > C sites.

Loci	Genotype	No.	Genotype frequency	Allele	Allele frequency	Ne	Ho	He	PIC	HWE *p* Values
IGFALS g.918 G > C	CC	987	0.73	C	0.845	1.355	0.738	0.262	0.228	0.082
	CG	315	0.23							
	GG	51	0.04	G	0.155					

*Note*: Ne: effective allele number; He: expected heterozygosity; Ho: expected homozygosity; PIC: polymorphism information content; HWE: Hardy–Weinberg equilibrium.

### Statistical associations between genotypes and the studied traits

In a statistical analysis of genotypes and scrotal circumference at different stages of development, there was a significant association between individuals with the CC genotype and the CG, GG genotypes **(**Table 5**)**. The statistical results showed that the scrotal circumference of individuals with CC genotype was significantly lower than that of individuals with CG and GG genotype at 100, 120 and 180 days of age (*p* < 0.05). At 140 and 160 days of age, the scrotal circumference of individuals with CC genotype was significantly lower than that of individuals with CG genotype (*p* < 0.05).

**Table 5.** Association of different genotypes due to SNP in *IGFALS* with the scrotal circumference in sheep.

Item	*IGFALS* g.918 G > A			*p-*Value
	CC	CG	GG	
No	987	315	51	
Scrotal circumference100 (cm)	18.230 ± 0.104^b^	18.851 ± 0.185^a^	19.443 ± 0.459^a^	<0.01
Scrotal circumference120 (cm)	22.011 ± 0.119^b^	22.657 ± 0.210^a^	23.402 ± 0.523^a^	<0.01
Scrotal circumference140 (cm)	25.604 ± 0.105^b^	26.210 ± 0.187^a^	26.153 ± 0.464^ab^	<0.01
Scrotal circumference160 (cm)	26.901 ± 0.107^b^	27.424 ± 0.190^a^	27.663 ± 0.471^ab^	<0.01
Scrotal circumference180 (cm)	26.829 ± 0.113^b^	27.482 ± 0.200^a^	27.896 ± 0.497^a^	<0.01

*Note*: SNP: single nucleotide polymorphism. Vales for the phenotypic data are shown as the mean ± standard error. Different superscript lowercase letters against values in the same column indicate significant differences (*p* < 0.05).

## Discussion

Reasonable use of breeding rams can reduce production costs, shortens the generation interval and may increase economic benefits. Scrotal circumference is an important indicator of the reproductive capacity of rams. It has been shown that the larger the circumference of scrotum, the higher the sperm yield and the better the fertility effect.^36^ There are many factors that affect the size of the scrotal circumference. Studies have found that there are significant differences in the biological and endocrine characteristics of the scrotum and testes among bulls of different ages in the same season. Similarly, there are significant differences in the scrotal circumference and endocrine characteristics among bulls of the same age in different seasons.^37^ In addition to age and season, there are also factors such as body weight^38^ and nutrition.^39^^,^^40^ In this study, 1353 Hu sheep were measured for scrotal circumference at different developmental stages under the same level of feeding management. Through statistical analysis, the results showed that the scrotal circumference increases with age from 100 to 160 days old, but remained unchanged at 160 and 180 days old. The coefficient of variation of scrotal circumference at each stage was greater than 10%, indicating that there was a certain selection space for this trait. In addition, we estimated the heritability of scrotal circumference at each stage, and the results showed that the heritability of scrotal circumference at each stage ranged from medium to high, ranging from 0.318 to 0.719. In the study of Rambouillet rams by Matos et al.,^41^ it was found that the circumference of the scrotum increased with age from 90 to 180 days old, and the heritability at each stage was 0.22–0.60, indicating a moderate to high heritability, which is consistent with the results of this study. In this study, there was no change in the scrotal circumference at the ages of 160 and 180 days, as the growth of Hu sheep began to slow down from 3 to 6 months old, and the scrotal circumference was also related to body weight. Therefore, there was no change in the scrotal circumference at the ages of 160 and 180 days.

The size of scrotal circumference, as an important basis for judging the quality of breeding rams, will directly or indirectly affect the efficiency of sheep breeding industry. Screening the genetic variation of key candidate genes related to scrotal circumference will help to better formulate breeding planning. *IGFALS* (Insulin-like growth factor-binding protein acid-labile subunit) genes play an important role in regulating growth and development and other physiological processes.^28^ In this study, the *IGFALS* gene was used as a candidate gene to investigate the relationship between its genetic variation and scrotal circumference. By DNA sequencing, a synonymous mutation (g.918 G > C) was detected in exon 1 of the *IGFALS* gene. Although synonymous mutation will not change the amino acid sequence of the protein encoded by the gene, it can affect its function by affecting cellular processes such as mRNA stability,^42^ mRNA translation,^43^ mRNA folding^44^ or splicing,^45^ protein folding^46^ and so on. In addition, some studies have found that synonymous mutations in non-coding regions are highly correlated with some important economic traits of sheep.^47^^,^^48^ The results of association analysis showed that the synonymous mutation g.918G > C significantly affected the scrotal circumference in Hu sheep. The mutation at 918 locus of sheep *IGFALS* gene in our study did not change the amino acid sequence of the protein. As previously reported, synonymous mutation may cause the change of protein function through the processes of mRNA stability^42^ and splicing events,^49^ because GG genotype had a higher phenotype value in scrotal circumference. Secondly, the development of the scrotum is a complex process, which is affected by multiple regulatory mechanisms. The cellular processing, proliferation, differentiation and prevention of apoptosis in sheep are affected by growth factors, in which insulin growth factors I and II (IGFs) play an important role.^50^ The protein ALS encoded by Insulin-like growth factor-binding protein acid-labile subunit (*IGFALS*) is an important compound, it connects IGF-I and IGFBP-3 to form a ternary complex in the cycle to regulate growth, development and other physiological processes.^28^ A previous study identified two new recessive mutations in the *IGFALS* gene.^51^ The N276S mutation led to changes in conserved amino acids, and the Q320X mutation caused severe truncation of the ALS protein. They concluded that the initial deficiency of ALS caused by *IGFALS* mutation reduced the formation of triple complex, thereby reducing growth after birth in these patients. In this study, we estimated the genetic parameters of scrotal circumference in five growth stages of Hu sheep and screened a molecular marker (*IGFALS* g.918 G > C) related to scrotal circumference in sheep, which is the advantage of this study. Unfortunately, this study did not Conjoint analysis the relationship between scrotal circumference, body weight and semen quality, and did not verify the level of cell function. Therefore, future research will involve Conjoint analysis of scrotal circumference, body weight and semen quality, Functional verification at the cell level, and verification between different varieties.

## Conclusions

In this study, the measurement of scrotal circumference at different stages, descriptive statistics and heritability estimation found that the scrotal circumference of Hu sheep aged 100 to 160 days increased with age, while there was no change from 160 to 180 days old. And the coefficient of variation of scrotal circumference at each stage exceeds 10%, with heritability ranging from 0.318 to 0.719, indicating a moderate to high heritability. This indicates that scrotal circumference as an important indicator for evaluating sheep’s reproductive ability has great potential for selection. In addition, a polymorphism g.918 G > C was detected in the *IGFALS* gene, which is associated with sheep scrotal circumference. Association analysis showed that individuals with GG and CG genotypes have larger phenotypic values of scrotal circumference. It indicates that *IGFALS* gene may be a candidate gene for sheep scrotal development and has potential research value. But further research is still needed.
